# Diets with Higher ω-6/ω-3 Ratios Show Differences in Ceramides and Fatty Acid Levels Accompanied by Increased Amyloid-Beta in the Brains of Male APP/PS1 Transgenic Mice

**DOI:** 10.3390/ijms222010907

**Published:** 2021-10-09

**Authors:** Lara Ordóñez-Gutiérrez, Gemma Fábrias, Josefina Casas, Francisco Wandosell

**Affiliations:** 1 Centro de Investigación Biomédica en Red de Enfermedades Neurodegenerativas (CIBERNED), 28049 Madrid, Spain; lordoniez@cbm.csic.es; 2 Centro de Biología Molecular “Severo Ochoa” (CSIC-UAM), 28049 Madrid, Spain; 3 Instituto de Química Avanzada de Cataluña (IQAC-CSIC), 080034 Barcelona, Spain; gemma.fabrias@iqac.csic.es (G.F.); jcbqob@iqac.csic.es (J.C.)

**Keywords:** Alzheimer, neurodegeneration, amyloid, ω-6/ω-3, PUFA, ceramides

## Abstract

Senile plaque formation as a consequence of amyloid-β peptide (Aβ) aggregation constitutes one of the main hallmarks of Alzheimer’s disease (AD). This pathology is characterized by synaptic alterations and cognitive impairment. In order to either prevent or revert it, different therapeutic approaches have been proposed, and some of them are focused on diet modification. Modification of the ω-6/ω-3 fatty acids (FA) ratio in diets has been proven to affect Aβ production and senile plaque formation in the hippocampus and cortex of female transgenic (TG) mice. In these diets, linoleic acid is the main contribution of ω-6 FA, whereas alpha-linoleic acid (ALA), eicosapentaenoic acid (EPA), docosahexaenoic acid (DHA) and docosapentaenoic acid (DPA) are the contributors of ω-3 FA. In the present work, we have explored the effect of ω-6/ω-3 ratio modifications in the diets of male double-transgenic APPswe/PS1ΔE9 (AD model) and wild-type mice (WT). Amyloid burden in the hippocampus increased in parallel with the increase in dietary ω-6/ω-3 ratio in TG male mice. In addition, there was a modification in the brain lipid profile proportional to the ω-6/ω-3 ratio of the diet. In particular, the higher the ω-6/ω-3 ratio, the lower the ceramides and higher the FAs, particularly docosatetraenoic acid. Modifications to the cortex lipid profile was mostly similar between TG and WT mice, except for gangliosides (higher levels in TG mice) and some ceramide species (lower levels in TG mice).

## 1. Introduction

Alzheimer’s Disease (AD), one of the most important neurodegenerative diseases, is characterized by the presence of senile plaques and neurofibrillary tangles in many brain regions [1]. In AD, there is a progressive loss of neurons in hippocampal and cortical areas, which finally impair cognitive function [2]. It has been described that processing amyloid precursor protein (APP) through the amyloidogenic pathway produces amyloid-β peptide (Aβ), which accumulates in the brain to produce senile plaques [2]. According to the amyloidogenic theory, Aβ-induced toxicity results in a series of events, which ultimately results in oxidative stress, mitochondrial dysfunction and synaptic impairment [3,4]. A small percentage of cases have a genetic origin and are known as Familial AD (FAD), caused by mutations in APP [5], presenilin 1 [6] or presenilin 2 [7] genes. However, more than 98% of AD cases are sporadic (unknown cause) with relation to some risk factors, aging being the most important. Environmental and/or dietary factors may also have a strong influence on the appearance of this disease, and increasing evidence suggests an association between AD and certain metabolic disorders including diabetes or hypercholesterolemia [8,9,10,11]. In human populations, some reports have shown a clear decrease in the risk of suffering AD and other dementias in people who consume large quantities of fish containing high levels of unsaturated fatty acids, which have a small ω-6/ω-3 lipid ratio [12,13].

The first outline relating lipids and AD dates back to Alois Alzheimer, who noticed lipid granule accumulation in the glia surrounding amyloid plaques [1]. Cerebral lipid peroxidation was found to be an early event in AD. The brains of AD patients display a higher number of lipoid granules (or adipose inclusions) in the glia, suggesting aberrant lipid metabolism [14]. Increasing data describe alterations in sphingolipid metabolism in AD. Modification in the levels of ceramides and sphingomyelins were observed in plasma and brain samples of AD patients. Moreover, AD animal models showed altered levels of ceramides, gangliosides, phospholipids, sulfatides and sphingomyelins. In fact, sphingolipids are targeted in new therapeutic approaches [15].

Other important lipids, fatty acids, consist of a carbon backbone that terminates in a carboxylic acid functional group, and they are categorized into different subclasses based on the length of the carbon chain. Depending on the number of double bonds, fatty acids are classified as monounsaturated (MUFA) or polyunsaturated (PUFA). PUFAs include the essential fatty acids ω6-linoleic acid (LA) and ω3-alpha-linolenic acid (ALA). While the brain can synthetize the majority of required saturated and monounsaturated fatty acids, it lacks the ability to synthesize essential PUFAs [16]. ALA is metabolized to eicosapentaenoic acid (C20:5 ω-3, EPA) and subsequently to docosahexaenoic acid (DHA), while LA is the precursor of arachidonic acid (AA) [17]. In the synthesis of ω-3 and ω-6 PUFA, there is a complex enzymatic process that allows the desaturation and elongation of the 18-carbon precursors (ALA and LA). The responsible enzyme showed a higher affinity for ω-3 PUFA than for ω-6 PUFA, favoring the transformation of ALA into DHA [18]. This different affinity inspired some recommendations (5:1 molar ratio) for the dietary intake of ω-6/ω-3 PUFA. However, the Food and Agriculture Organization of the United Nations supported that there is no rationale to specifically recommending an ω-6 to ω-3 ratio or LA to ALA ratio if intakes of ω-6 and ω-3 fatty acids occur in the normal diet [19].

ALA is found in higher amounts in vegetable oils (sunflower, soybean or corn), while the principal dietary sources of preformed DHA include blue fish (salmon, tuna, anchovy or sardine). This is due to the fact that food for many fish includes algae or marine invertebrates rich in EPA and DHA. In contrast, the main dietary sources of AA are foods of animal origin such as beef, pork, lamb, chicken, turkey and eggs [18,20]. 

DHA appears to have been conserved in neural signaling systems during animal evolution, and it is still the major functional and only component responsible for vision, all sensory and motor functions, and cognition, as well as participating in the control of blood flow and neural gene expression [21].

Diets enriched in PUFAs have also been associated with a lower incidence of dementia and neurological disorders [22], and diets containing low percentage of DHA (22:6 ω-3) and ω-3 PUFAs might be linked to cognitive impairment [23,24]. Furthermore, the brain pathological phenotype and cognitive effects of apoE4 were reduced by a high DHA diet and accentuated by high cholesterol [25]. It has been reported that the administration of high-DHA diets reduced Aβ levels in several transgenic (TG) mouse models including Tg2576 [26], APPswe/PS1ΔE9 [27] and 3xTg-AD [28]. More recently, we described that PUFA-content-modified diets differentially altered the amyloid burden and lipid composition in the brain depending on the hormonal status in females [29]. Sexual differences have been described regarding microglial localization and morphology [30] and in the response to palmitic acid [31] or saturated fatty acids [32,33]. However, many reports lack the comparison between males and females.

Given the results observed in wild-type (WT) [34] and AD TG females [29], we consider it essential to analyze the effect of diet on males. The purpose of this study was to determine the effect of different ω-6/ω-3 ratios in the diet of male double-transgenic APPswe/PS1ΔE9 and WT mice. At the end point, an analysis of the brain cortex lipid profile of these animals was performed, and the levels of human Aβ_1-40_ and Aβ_1-42_ were measured in TG mice hippocampi. Some protein markers were verified to address the effect of the diets on pathological and synaptic pathways.

## 2. Results 

### 2.1. Diet Composition

The composition of the three diets was analyzed by UpScience (Vannes, France) and presented summarized in Table 1 and is more complete in Supplementary Table S1. The total fat content was 4.6 g/100g in SF, 7.3 g/100g in H and 6.9 g/100g in L. There were no significant differences between the three diets in the level of saturated or unsaturated fatty acids or in the MUFA and PUFA content (Table 1).

Differences became evident when comparing several relevant lipid species, such as the ω-3 PUFAs C18:3ω-3 (ALA), C20:5ω-3 (EPA), C22:5ω-3 (DPA) and C22:6ω-3 (DHA). Of these, only ALA was detected in the SF diet, whereas EPA and docosapentanoic acid (DPA) were not detected in the H diet. The greatest ω-3 content was measured in the L diet (615 mg/100g), underscoring the levels of DHA (177 mg/100g), EPA (108 mg/100g) and ALA (193 mg/100g). For the remainder of this article, the diets will be represented and ranked in increasing order depending on the ω-6/ω-3 ratio: L (ω-6/ω-3 = 3.2); SF (ω-6/ω-3 = 12.3); and H (ω-6/ω-3 =24.6).

### 2.2. Effect of Diet on Body Weight

**Figure 1:**
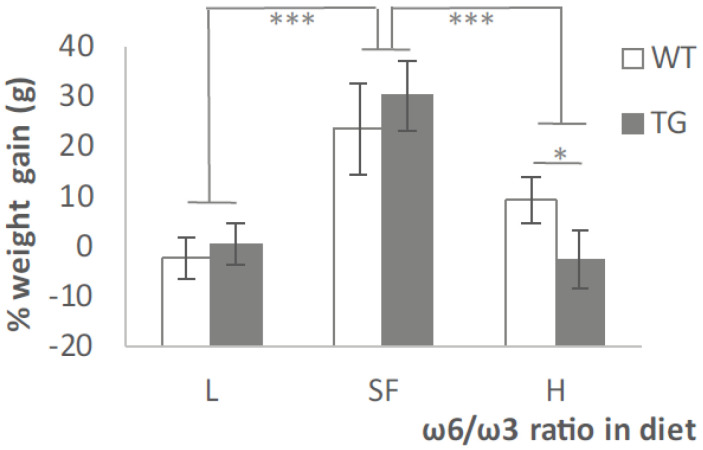
Changes in body weight after three months of modified diet. Results are shown as the medium ± SEM of the percentage of weight gained from 3 to 6 months of age. *: *p* < 0.05; ***: *p* < 0.001.

At 3 months of age, groups of TG or WT mice were switched from normal diet (SF) to a diet containing lower or higher ω-6/ω-3 ratio (L or H, respectively) and weighed weekly for 3 months. The percentage of the weight gained in this period (from 3 to 6 months-of-age) is represented in Figure 1. WT and TG mice fed with L diet maintained mostly the same weight during these 3 months. H diet produced a 10% increase in the weight of WT mice in contrast to TG mice that showed lighter reduction (*p* < 0.05). WT and TG mice fed the SF diet grew by 20% and 30%, respectively (*p* < 0.001). Although these differences were statistically significant, the variations in weight were not meaningful enough to be considered as a substantial dietary side effect.

### 2.3. Effect of Diet on the Brain Lipidome

After 3 months of being fed the H, L or SF diets, the WT and TG mice were sacrificed, and cortex samples were collected for analysis of several lipid species in the brain. We focused our analysis on fatty acids, phospholipids and certain sphingolipids: ceramides, sphingomyelins, gangliosides and sulfatides. 

**Figure 2:**
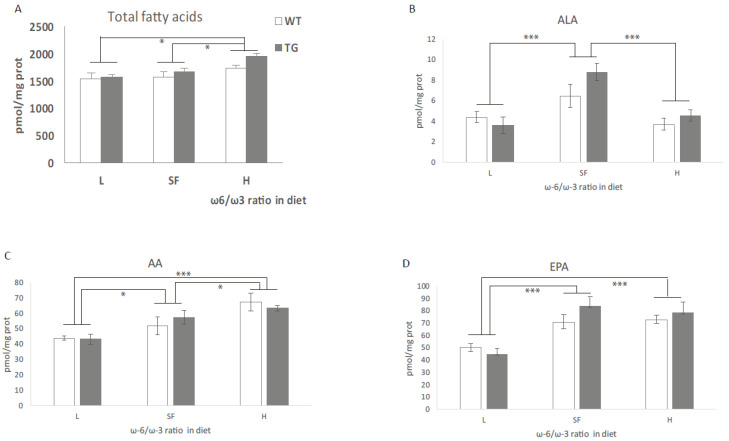

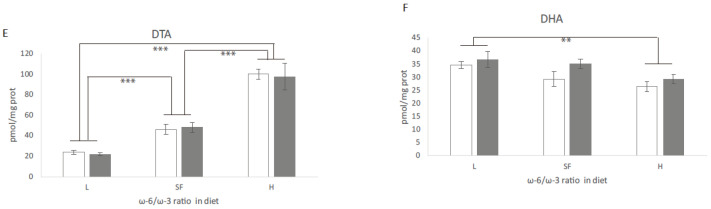
(**A**) Total fatty acid (FA), (**B**) alpha-linolenic acid (ALA), (**C**) arachidonic acid (AA), (**D**) eicosapentaenoic acid (EPA), (**E**) docosatetraenoic acid (DTA) and (**F**) docosahexaenoic acid (DHA) levels in the brains of transgenic (TG) and wild-type (WT) male mice after being fed with L, SF and H diets for 3 months. Results are shown as the medium ± SEM and represented with respect to ω-6/ω-3 ratio in the diet. *: *p* < 0.05; **: *p* < 0.01; ***: *p* < 0.001. L = lower ω-6/ω-3 ratio in the diet; H = higher ω-6/ω-3 ratio in the diet; SF = standard food.

There were no differences observed in total fatty acids between TG and WT mice (Figure 2A), with both genotypes increasing in parallel corresponding to the higher ω-6/ω-3 content. 

Some important PUFAs, ω-3 and ω-6 were measured (Figure 2). In general, an increase in AA, EPA and DTA was evident, in parallel to an increase in ω-6/ω-3 ratio in the diet. The most significant increase (5-fold) was observed in the levels of DTA (Figure 2E). The opposite effect was observed on the levels of DHA, which decreased in parallel to the increase in ω-6/ω-3 ratio (Figure 2F). As expected, the H diet (with the lowest DHA content) produced a significant reduction in DHA levels in the cortex. DPA levels were not modified by any of the diets (data not shown). 

Despite the changes observed in total fatty acids after H diet, we also represented the levels of EPA, DTA and DHA with respect to total fatty acids (Supplementary Figure S1), observing that the levels of DHA adjusted for total fatty acids were not modified between diets or genotypes.

**Figure 3:**
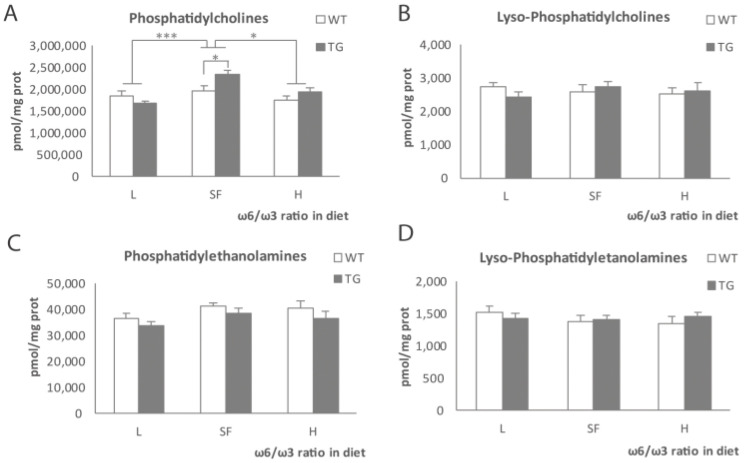
Phosphatidylcholines (**A**), lyso-phosphatidylcholines (**B**), phosphatidylethanolamines (**C**) and lyso-phosphatidyl-ethanolamines (**D**) levels in the brains of transgenic (TG) and wild-type (WT) male mice after being fed with L, SF and H diets for 3 months. Represented with respect to ω-6/ω-3 ratio in the diet. Results are shown as the medium ± SEM. *: *p* < 0.05; ***: *p* < 0.001. L = lower ω-6/ω-3 ratio in the diet; H = higher ω-6/ω-3 ratio in the diet; SF = standard food.

After analysis of the fatty acids, we studied the levels of certain phospholipids in the cortex of the TG and WT male mice fed with the three diets. Phosphatidylcholine (Figure 3A) levels showed differences between diets in the TG but not WT mice. TG mice fed with H or L diets showed a reduction in phosphatidylcholine when compared to the SF group (*p* < 0.001 and *p* = 0.024, respectively). Differences between genotypes were statistically significant in the SF-fed group (*p* = 0.015) but not in the other two dietary groups. Meanwhile, the differences between diets were not statistically significant in the analysis of lyso-phosphatidylcholines (Figure 3B), phosphatidyl-ethanolamines (Figure 3C) and lyso-phosphatidyl-ethanolamines (Figure 3D), regardless of genotype or diet.

**Figure 4:**
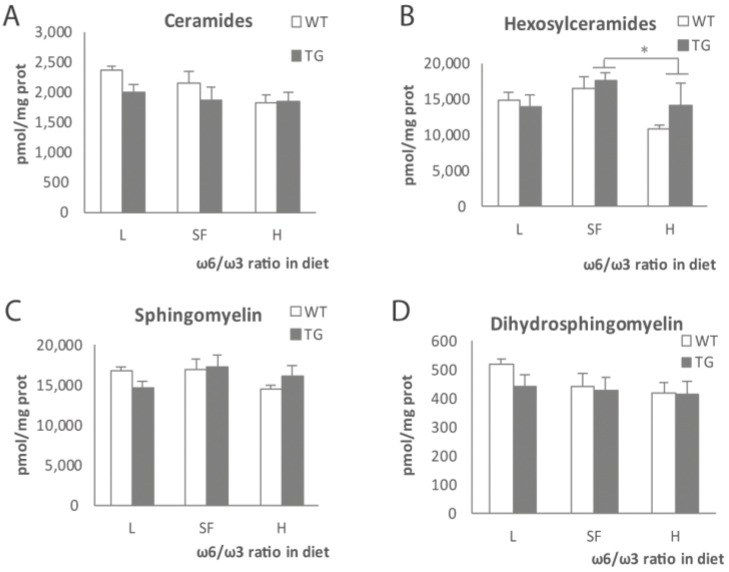
Total ceramides (**A**), hexosylceramides (**B**), sphingomyelins (**C**) and dihydrosphingomyelins (**D**) levels in the brains of transgenic (TG) and wild-type (WT) male mice after being fed with L, SF and H diets for 3 months. Represented with respect to ω-6/ω-3 ratio in the diet. Results are shown as the medium ± SEM. *: *p* < 0.05. L = lower ω-6/ω-3 ratio in the diet; H = higher ω-6/ω-3 ratio in the diet; SF = standard food.

**Figure 5:**
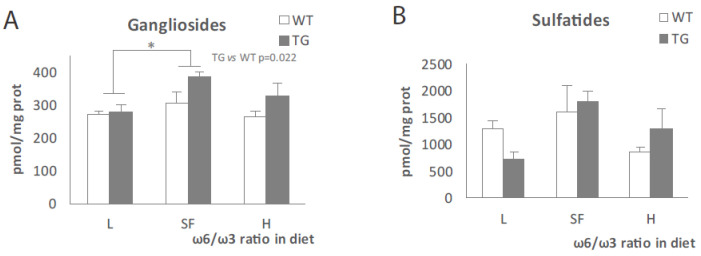
Gangliosides (**A**) and sulfatides (**B**) levels in the brains of transgenic (TG) and wild-type (WT) male mice after being fed with L, SF and H diets for 3 months. Represented with respect to ω-6/ω-3 ratio in the diet. Results are shown as the medium ± SEM. *: *p* < 0.05. L = lower ω-6/ω-3 ratio in the diet; H = higher ω-6/ω-3 ratio in the diet; SF = standard food.

Next, we moved to check some members of the sphingolipid family: ceramides and sphingomyelins (Figure 4) and gangliosides and sulfatides (Figure 5). Total ceramides showed a tendency to decrease in parallel with the increase in ω-6/ω-3 ratio in diet (Figure 4A), and although more evident in the case of WT mice, it was not statistically significant in any genotype. In the case of hexosylceramides (Figure 4B), we observed a significant reduction in the H diet group when compared to the SF group (*p* = 0.045) in both genotypes. The analysis of sphingomyelins (Figure 4C) and dihydrosphingomyelins (Figure 4D) did not show statistically significant differences between groups regardless of genotype. 

**Figure 6:**
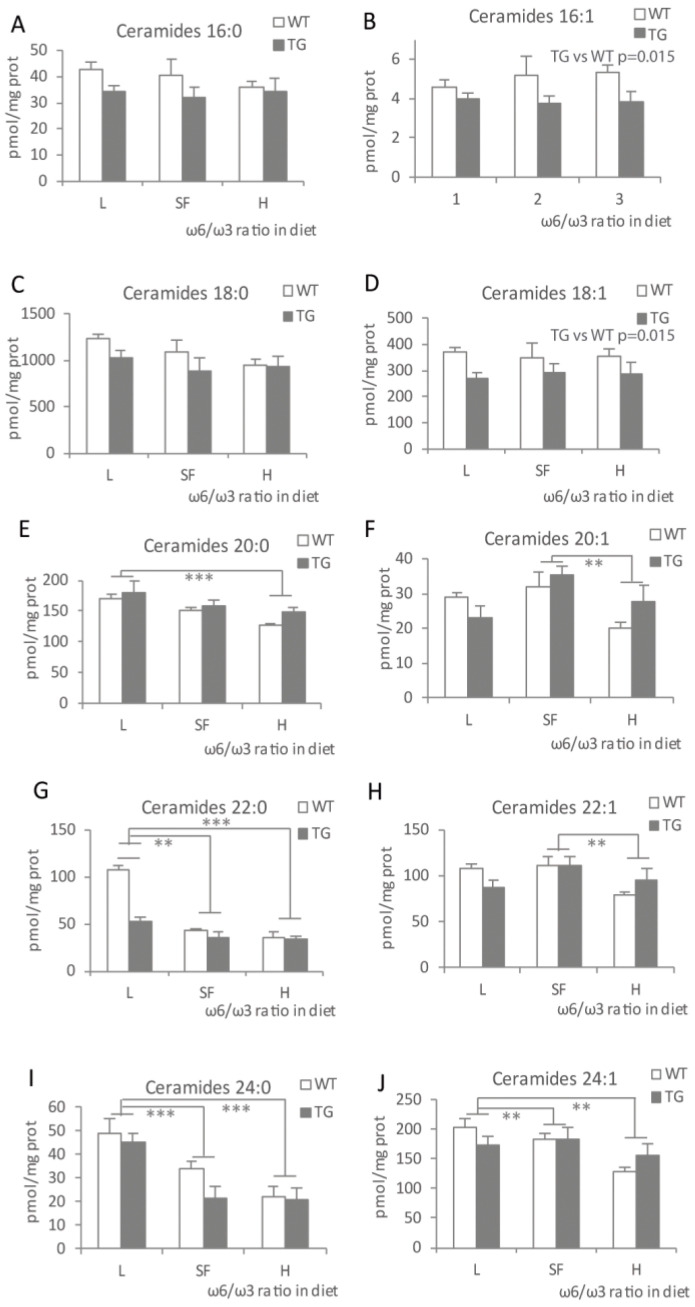
Levels of selected ceramide species in the brains of transgenic (TG) and wild-type (WT) male mice after being fed with L, SF and H diets for 3 months. Ceramides 16:0 (**A**), 16:1 (**B**), 18:0 (**C**), 18:1 (**D**), 20:0 (**E**), 20:1 (**F**), 22:0 (**G**), 22:1 (**H**), 24:0 (**I**) and 24:1 (**J**). Represented with respect to ω-6/ω-3 ratio in the diet. Results are shown as the medium ± SEM. **: *p* < 0.01; ***: *p* < 0.001. L = lower ω-6/ω-3 ratio in the diet; H = higher ω-6/ω-3 ratio in the diet; SF = standard food.

When looking at gangliosides C18:1 (Figure 5A), both L and H diets produced a reduction when compared to the SF diet, which was statistically significant in the case of the L diet (*p* = 0.021). Ganglioside levels were higher in TG mice (*p* = 0.022) compared to WT. Sulfatide levels tend to be lower (Figure 5B) in the H and L diet groups when compared to SF-fed animals but not statistically significant (*p* = 0.07 and *p* = 0.06, respectively), both in TG and WT mice. Since some specific ceramides have been described to be altered in TG mice and AD patients, we decided to pursue a deeper analysis. In general, most ceramide species showed a tendency to decrease in line with increasing dietary ω-6/ω-3 ratio (Figure 6), although not all reached statistical significance. Ceramides C16:1 and C18:1 were higher in WT mice (*p* = 0.015) fed with any of the three diets. However, the amount of these ceramides did not vary by diet. The rest of the ceramide species tested showed decreasing levels in parallel with an increasing dietary ω-6/ω-3 ratio in both genotypes. Even though the results for total ceramides showed only a tendency (Figure 4A), the effect was significant for the specific ceramides C20:0, C20:1, C22:0, C22:1, C24:0 and C24:1 (Figure 6). The strongest effect was observed in the H-fed mice, with a clear reduction in most ceramide species compared to the other two diets.

### 2.4. Effect of Diet on Burden of Amyloid Deposition

**Figure 7:**
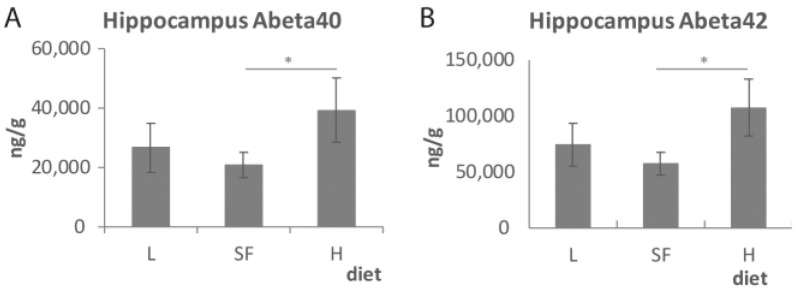
Aβ1_-40_ (**A**) and Aβ1_-42_ (**B**) levels in the hippocampus of transgenic (TG) male mice after being fed with L, SF and H diets for 3 months. Represented with respect to ω-6/ω-3 ratio in the diet. Results are shown as the medium ± SEM. *: *p* < 0.05. L = lower ω-6/ω-3 ratio in the diet; H = higher ω-6/ω-3 ratio in the diet; SF = standard food.

In order to determine whether different diets may alter the normal amyloid accumulation in the hippocampi of the TG male mice, we measured the levels of human Aβ1_-40_ and Aβ1_-42_ by ELISA (Figure 7). Both Aβ1_-40_ and Aβ1_-42_ showed a similar pattern, with a significant increase provoked by 3 months of H diet administration compared to SF which is commonly used in our animal facilities (*p* = 0.03 and *p* = 0.01 for Aβ1_-40_ and Aβ1_-42_, respectively). The amyloid levels observed in mice fed with diets L and SF were not significantly different. These results indicate that the lowest ω-3 PUFA content in the diet exacerbated the Aβ accumulation.

### 2.5. Effect of Diet on Brain Protein Markers

In order to further understand the effects of the three diets, homogenized cortex samples were analyzed by Western blot to check the levels of different protein markers. Initially, we analyzed the levels of different pre-synaptic and post-synaptic markers such as P120, PSD95, phospho-synapsin and synaptophysin. These synaptic proteins have been described to be altered in human neurodegenerative disorders and in TG models, including the APP/PS1 strain used in this study. Additionally, we measured the levels of some markers linked to amyloid pathology such as APP, BACE, GFAP and tau (PHF1). APP (precursor) and BACE (processing enzyme) are directly related to the production of amyloid peptide, of which increased levels are known to provoke an increase in phosphorylated tau, which was measured with the PHF1 antibody. This environment produces an inflammatory process characterized by the presence of activated glial cells. This effect has been studied analyzing the level of activated astrocytes using GFAP antibody. All antibodies used are summarized in Supplementary Table S2. Some proteins were analyzed in TG and WT mice fed with SF in order to standardized possible differences caused by the genotype (Supplementary Figure S2). As expected, we found differences in APP and BACE between TG and WT mice. The differences in the other proteins were not statistically significant.

**Figure 8:**
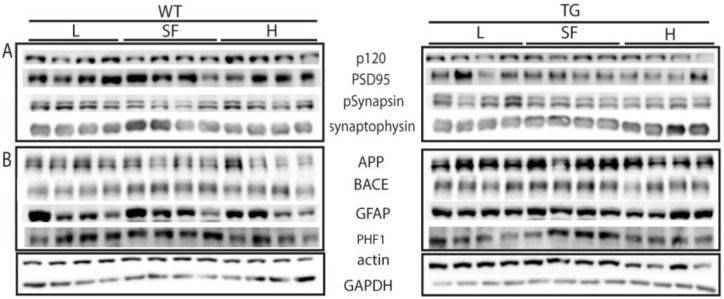
Synaptic (**A**) and pathology (**B**) markers in the cortex of transgenic (TG) and wild-type (WT) male mice after being fed with L, SF and H diets for 3 months. L = lower ω-6/ω-3 ratio in the diet; H = higher ω-6/ω-3 ratio in the diet; SF = standard food.

In summary, the effect of the diets on protein markers was not evident at all. Slight differences observed in some Western blot membranes were not statistically significant when we summarized all the analyzed data (Figure 8). 

The levels of synaptic markers were slightly modified by the increase or reduction in ω-6/ω-3 ratio in the diet in both TG and WT mice. PSD95 seemed to be reduced by H diet in WT mice, whereas the effect on TG mice was not so evident. Phospho-synapsin protein showed a tendency to increase in both WT and TG mice fed with H diet. A similar pattern was observed in the levels of synaptophysin of TG groups. 

When we analyzed the levels of some proteins that could be altered because of the pathology, we observed reduced levels of APP in WT mice fed with H diet. This effect was not observed in TG mice, but it must be considered that the overexpression of APP in TG may affect this symptom. The other protein that participates in Aβ production, BACE, was not evidently affected by the alteration in DHA levels in the diet either. A similar situation occurs when we analyzed the levels of glial activated cells by the GFAP or the neuronal phosphorylated tau proteins by PHF1.

## 3. Discussion

Previous studies have related dietary ω-6/ω-3 ratio to neuroprotective effects such as prevention of neurodegeneration and cognitive impairment [35,36]. The consumption of fish oil and ω-3 fatty acids has been directly linked to brain health and a lower risk of development of AD [12,37]. 

Dietary habits, gender and APOE4 genotype are considered important when designing new dietary modifications based on increased levels of ω-3 PUFAs. The plasma levels of EPA and DHA positively correlated with a higher frequency of fish consumption in [38], a study which also showed different levels of EPA in women when compared to men. This sex effect has also been observed in TG mice [39] and even in the response of both sexes to high fat diets [40]. However, how diet and sexual hormones interacts in pathological situations such as AD is not fully understood.

The present study tested the effect of three different ω-6/ω-3 ratios in the diet of TG and WT male mice. Interestingly, the data show that diets containing lower levels of DHA and higher ω-6/ω-3 ratio produced an increase in the amyloid burden in TG male mice. However, considering that the total amount of ω-6 between H and L diet is not so important (Table 1), we could hypothesize that the increased Aβ levels in males fed with H diet correlated with lower ω-3 content (H diet). Previous data, already published that ω-3 FA improved Aβ phagocytosis in human microglia [41,42]. Then, reduced ω-3 FA in the diet could be associated to increased Aβ levels that microglia perhaps mediated. In fact, we did not detect differences in astrocyte marker GFAP, which strongly support that astrocytes were not affected along with treatment in TG or WT. 

We have to remind the reader that our previous studies performed on females showed that increased ω-6/ω-3 ratios reduced amyloid levels in the absence of estrogen. Meanwhile, the opposite effect was observed in the presence of estrogens, and an increase in amyloid brain burden in parallel with the increase in ω-6/ω-3 ratio in the diet was observed [29]. Thus, an increase in ω-6/ω-3 ratio produced an increase in amyloid levels both in male and females in the presence of the physiological amount of sexual hormones.

However, ovariectomized females (hence, with reduced estrogen levels) showed reduced levels of amyloid accumulation in the brain after being fed a diet with higher ω-6/ω-3 ratio. Surprisingly, males were not sensitive to testosterone depletion by castration (Supplementary Figure S3); thus, these differences in the sensitivity between males and females are remarkable. 

This different sensitivity to dietary modification observed between males and females has been described in several reports using high fat diet [43] or several human diet types [44] such as ketogenic, Japanese or Mediterranean. The effect of estrogens has been studied in relation to dietary response, and some sex differences have been described. In fact, estrogens have been shown to protect against obesity and high fat intake by modifying ER alpha receptor in the hypothalamus of males but not females [29]. 

Additionally, diets enriched specifically DHA have been shown to reduce amyloid pathology in TG mouse models of AD [26,27,28]. Compared to our previous results [29,34], we observed similar patterns in males and females with reduced DHA levels in the animals fed with the highest ω-6/ω-3 ratio. Meanwhile, these differences were masked when DHA levels are represented with respect to total fatty acids. Even though the level of DHA in SF pellets was not detectable, the final impact on the DHA present in the brain was indistinguishable from the diet with higher DHA. This strongly suggests that the intake of DHA in the diet is not directly reflected in the amount of DHA in the brain, which might be derived from another source or be at least in part synthetized in brain cells.

The direct relationship between ω-3 FA in the diet and the evolution of neurodegenerative pathology is widely recognized. DHA and EPA have been described to stimulate macrophage [45] and microglial phagocytosis of Aβ and decrease inflammatory markers [46]. Moreover, high DHA-content diet increased the relative cerebral blood volume in TG mice [47]. Even so, the level of ω-6 FA is another critical factor [24]; since ω-6 and ω-3 fatty acids are competitive at many enzymatic steps, the ω-6/ω-3 ratio must be balanced. On the other hand, reduced levels of ω-3 FA in the diet have been linked to increased risk of developing neurodegenerative diseases in rodent models and humans [23,44,48,49]. However, some clinical trials could not conclude a clear link [50,51].

Ceramides are the core constituents of most synthetized sphingolipids, and they can be produced either by hydrolysis of sphingomyelin via sphingomyelinases or synthesized de novo from palmitoyl-CoA and serine [52]. In this study, male mice showed similar ceramide levels when compared by genotype (TG and WT) or diets (H, L and SF). A similar unchanged pattern was observed in the study of sphingomyelin levels in male mice. However, it is important to remember that the diets contained increasing amounts of palmitic acid (SF > H > L), without any apparent effect on the final brain content.

This is in contrast to the remarkable differences that we previously observed in females [29,34], which were very sensitive to lipid modification of the diet; the low ω-6/ω-3 ratio diet produced a five-fold increase in ceramides WT and two-fold in TG females. However, the palmitic acid present in the diet in these experiments was more abundant (quantity in L diet was the highest; meanwhile, levels in SF and H diet were similar) [29,34]. 

Ceramide plays an important role in the cellular signaling of processes including cell growth, differentiation and apoptosis in nearly every cell type. In the brain, ceramide stabilizes the APP-cleaving enzyme 1 (BACE1), thereby promoting Aβ biogenesis [53,54]. Thus, it has been suggested that ceramide and Aβ may synergize to induce neuronal death in AD; an increase in ceramide levels has been reported in the plasma and brains of AD patients compared to controls [55,56,57]. However, differences observed between patients could be influenced by different diets. Some ceramides (Cer(d18:1/18:0) and Cer(d18:1/24:0)) have been described to accumulate in gray matter [58] and in lipid rafts of AD patients [59]. Elevated levels of ceramides C16, C18, C20 and C24 were found in brains from patients with AD and other neuropathological abnormalities [56]. In addition, senile plaques have been described to be enriched in saturated ceramides Cer (d18:1/18:0) and Cer (d18:1/20:0) [52]. 

From the six mammalian Cer synthases (CERS) described, CERS1 is expressed in brain, especially in neurons, and exhibits increased activity toward C18-CoA, whereas CERS2 prefers C22-CoAs and C24-CoAs as substrates [60,61,62]. Thus, it is tantalizing to propose that CERS1 is more affected by the genotype and CERS2 by the diet. 

Several previous publications have described the effect of lipid-diet modification on lipid brain profile, in particular, on ceramide content. Fish oil-enriched diet modified ceramide in the rat hippocampus [63]. Moreover, AD mouse models fed with sea cucumber (enriched in glucocerebrosides) showed altered levels of ceramides and sulfatides [64]. Dietary sphingomyelin supplementation affects cerebroside content and myelin development [65].

Ceramides are the main source of different sphingolipid series, such as gangliosides, sulfatides, lactosylceramides, etc. Gangliosides and sulfatides are essential for the maturation and maintenance of the nervous system, and mutations in genes involved in the ganglioside biosynthetic pathway result in neurodegenerative disorders in mice [66]. In our study, ganglioside levels in SF-fed TG male mice were higher, an effect which was almost abolished when they were fed with the L diet. In contrast, sulfatides maintained a tendency to be higher after feeding the SF diet in both genotypes. Similarly, the analysis of phosphatidylcholines showed higher levels in TG male mice fed with SF. An increased level of gangliosides and phosphatidylcholines has already been described in TG mice [67,68], although levels of both appear to be reduced in AD patients [69,70]. This apparent contradiction may be due to the fact that human brain samples mostly correspond to advanced AD patients, whereas some experiments were performed in adults in mouse models (6–10 month-old mice); this fact underlines the limitations of mouse models in the research of neurodegenerative diseases. Of course, our results can hardly be extrapolated into humans. The human metabolic process is slower than that of rodents, so lower levels of ALA as a precursor of DHA could be expected for humans. In contrast, administration of AA or DHA would be more effective [71].

Synaptic alterations have been characterized in depth in the APP/PS1 mouse model, however, they are usually more evident in aged mice. Decreased levels of synaptic markers such as PSD95, synaptophysin and synapsin have been described in older TG mice compared to WT [72,73,74]; however, analysis in younger mice did not show significant differences between them [75,76]. The mice analyzed in this study were sacrificed at 6 months of age and would still be considered young mice. At this age point, it is not so clear that the pathology would provoke significant alteration of synaptic markers, so the effect observed should be only related to the dietary effect. DHA has been described to increase synaptic proteins such as synaptophysin and PSD95 [77]. Our results showed no evident modifications of the analyzed proteins, although the levels of phospho-synapsin were strongly affected by lower dietary ω-6/ω-3 ratio in females [29,34]. This fact highlights the lower sensitivity of males when compared to females both in WT and TG animals. Obviously, we cannot discard that in males more time is needed to observe the data reported in females. 

This study was performed in a very early state of the disease where we could find amyloid deposition, but it lacks sufficient neurodegenerative processes to affect synaptic activity, as observed by Western blot. Thus, it is really important to confirm the effect of the diet as an anticipatory strategy, a manner to act before any symptoms are evident. Even in males, with a lower sensitivity to the diet modification, it was clear that reduced levels of DHA in the diet (higher ω-6/ω-3 ratio) increased the Aβ accumulation in the hippocampus.

All these data indicate that the response of females and males to the lipid modification of the diet differs significantly. In males, the differences in most of the sphingolipids analyzed between WT and APP were almost negligible except in some specific ceramides and sulfatides. In contrast, in most of the sphingolipids analyzed in females, the difference between WT and APP in some diets was more than evident [29]. 

A similar case happens at the protein level where we almost did not detect any major differences in males when we analyzed synaptic proteins or neuropathological markers such as Tau-phosphorylation. It is possible that we need to analyze older mice with an advanced state of the disease with more evident neurodegenerative features in order to observe the differences observed in APP females. Obviously, we cannot discard the possibility that the small differences in formulation of the DI (females) and H (males) diets may have a major impact. However, with respect to the other two diets tested, the differences were not so evident. 

In contrast, we observed that increasing levels of ω-6/ω-3 ratio produced higher amyloid accumulation in both sexes, although this effect was more evident in the case of females [29]. Thus, it is tantalizing to propose that the increase in amyloidosis might correlate of with lower ω-3 content in the diet.

Thus, we can hypothesize that males are more resistant to the effects of altered lipid content in the diet, making them stable in response to diet, whereas the plasticity previously observed in females allows for modulation of brain chemistry through the diet. However, it is important to remember that all the diets tested should be considered non-high fat diets, because the total amount of lipid was maintained at similar level to normal diet SF.

These results highlight the impact of the diet on the lipid profile in the brain and as a consequence on the evolution of neurodegenerative pathologies. In addition, differences found between males and females extol the importance of considering sexes separately in the study of AD and design of diet modification therapeutic strategies.

## 4. Materials and Methods

### 4.1. Animals and Husbandry 

Double-transgenic APP/PS1 mice (TG) were purchased from Jackson Laboratories (Bar Harbor; stock no. 005864). The strain B6.Cg-Tg (APPSwe, PSEN1dE9) 85Dbo/J overexpresses the human APP gene with the Swedish mutation and exon-9-deleted PSEN1. Mice were housed under constant temperature (22 ± 2 °C) and humidity (50 ± 5%), and a 12:12 h light–dark cycle in a specific-pathogen-free animal facility was applied. All animal care and handling strictly followed current Spanish legislation and guidelines and those of the European Commission (directive 2010/63/EU). All the procedures for use and management of the transgenic colony were approved by the Spanish Research Council (CEEA-CBMSO-33/307), the Community of Madrid (PROEX 341/15), which is recently extended by 5 years, the Spanish Research Council (CEEA-CBMSO-23/307.1), and the Community of Madrid (Ref. PROEX 069.7/21).

Pups were genotyped by polymerase chain reaction (PCR) analysis. Both genotypes (TG and WT) were used in these experiments; mice were allowed free access to food and tap water *ad libitum*. Starting from three months after birth, body weight was measured weekly. At this age, standard diet (SF) was gradually replaced and maintained until 6 months of age (endpoint), where they were then sacrificed by CO_2_ inhalation.

The orchiectomy procedure was performed following standard designed protocols [78]. 

### 4.2. Genotyping 

Pup genotype was confirmed by PCR analysis [79] using three primers: one antisense primer matching sequence within PrP (5′: GTG GAT ACC CCC TCC CCC AGC CTA GAC C), one sense primer specific for the transgene (PS1: 5_: CAG GTG GTG GAG CAA GAT G, APP: 5′: CCG AGA TCT CTG AAG TGA AGA TGG ATG), and a second sense primer specific for the genomic PrP (5′: CCT CTT TGT GAC TAT GTG GAC TGA TGT CGG). Only one band (prion (PrP) gene, used as internal control) was observed in the WT samples, whereas three bands (APP, PS1 and PrP) were observed in the TG samples.

### 4.3. Diet

SF (standard food) commonly used in our animal facilities was purchased from Scientific Animal Food and Engineering (France). Two experimental diets were purchased from PanLab (Spain) based on previous studies performed in females; however, the final composition was not identical. Thus, for the current study, we did not maintain previous nomenclature [29,34]. Three diets were subsequently analyzed by UpScience (France) to determine the final percentage of each fatty acid and the lipid profile (Supplementary Table S1).

The H diet (higher ω-6/ω-3 ratio) was modified with small amounts of ω-3 PUFAs containing sunflower oil (60 g/kg), oleic acid 18:1 ω-9 (10 g/kg) and with low DHA content (0.13 g/kg). This diet showed in the final analysis 34.5% in ω-6 PUFAs and 1.4% ω-3 PUFAs, with a resultant ω-6/ω-3 ratio of 24.6 (Table 1). This diet would be an example of a very low ω-3 PUFA content diet.

The L diet (lower ω-6/ω-3 ratio) was enriched with larger amounts of ω-3 PUFAs, containing less sunflower oil (30 g/kg) and more fish oil (40 g/kg). In this case, ω-6 PUFAs were 30.2% (similar to L diet); meanwhile, ω-3 PUFAs were 9.4% (more than 6-fold higher than H diet). This diet is an example of a fish-enriched diet, and in this case ω-3 FA are marine-derived ω-3 PUFAs. WT and TG male mice were fed with these diets for 90 days (from 3 to 6 months of age).

### 4.4. Tissue Processing

Mice were sacrificed at 6 months of age using CO_2_ inhalation, and their brains were snap frozen on dry ice for subsequent homogenization. A superficial necropsy was performed to test the general state of the organs (liver, spleen and kidney).

Brain tissue for lipidomic analysis was homogenized in 0.01% butyl-hydroxy-toluene (BHT) in PBS and stored in nitrogen atmosphere at −80 °C until analyzed. 

Brain tissue used for ELISA analysis was homogenized in eight volumes of ice-cold guanidine buffer, containing 5 M guanidine/HCl, 50 mM Tris HCl, pH 8. Homogenates were then mixed for 3 h at room temperature and stored at −20 °C until analyzed. 

Brain tissue used for Western blot analysis was homogenized in three volumes of ice-cold lysis buffer (20 mM HEPES, 100 mM NaCl, 100 mM NaF, 1 mM NaVO_4_, 5 mM EDTA and 1% Triton X-100) completed with protease inhibitor cocktail (Roche Diagnostic) and 1 μM okadaic acid (Calbiochem) as phosphatase inhibitor. Homogenate was centrifuged for 20 min at 4 °C at 16,000× *g*, and the supernatant with soluble proteins was stored at −80 °C. The protein concentration was measured using a DC Protein Assay (BioRad) following the manufacturer’s protocol. Proteins were resolved on an electrophoresis gel after the addition of loading buffer containing 10% sodium dodecyl sulfate (SDS), 0.5 mM Dithiothreitol, 250 mM Tris HCl, pH 6.8, 50% glycerol and bromophenol blue [80].

### 4.5. Lipid Analysis

The homogenized brain cortex was subjected to lipid extraction and analysis as previously described [81]. In all cases, the final data were calculated as pmol/mg of protein. Sphingolipids were analyzed by HPLC-MS (Liquid chromatography–mass spectrometry) [82] by using 0.2 nmol of C17-sphinganine, N-dodecanoyl-sphingosine, N-dodecanoyl-glucosyl-sphingosine and N-dodecanoyl-sphingosyl-phosphorylcholine as internal standards. Sphingolipids were annotated as <lipid subclass> <total fatty acyl chain length>:<total number of unsaturated bonds>. If the sphingoid base residue was dihydrosphingosine, the lipid class contained a <DH> prefix. 

### 4.6. Quantitative Determination of Aβ_1-40_ and Aβ_1-42_

For the determination of human Aβ_1-40_ and Aβ_1-42_ in hippocampus samples, two commercially available ELISA kits (Invitrogen, Burlington, MA, USA) were used. Briefly, brain homogenates were diluted 1:50 in PBS-Tween-BSA buffer (0.03% Tween-20, 5% BSA in PBS) before centrifugation (16,000× *g* for 20 min at 4 °C), and supernatants were analyzed immediately, following the manufacturer’s instructions. Plate absorbance was measured at 450 nm by using an Opsys MR microplate reader (Dynex Technologies, Chantilly, VA, USA) [80].

### 4.7. Western Blot 

Brain cortex tissue extracts were resolved by SDS-PAGE and transferred onto nitrocellulose (Amersham, Buckinghamshire, UK) or PVDF (Millipore, Burlington, MA, USA) membranes. Then, membranes were blocked by incubation with a 10% solution of non-fat milk for 1 h at room temperature. Primary antibodies (Supplementary Table S2) were incubated overnight at 4 °C, then washed in 0.1% Tween-PBS and incubated with the secondary horseradish peroxide-conjugated antibody (Supplementary Table S2). Antibody binding was detected with Western lighting^TM^ Plus ECL (Perkin-Elmer, Waltham, MA, USA) using β-actin, GAPDH or vinculin as internal loading controls. Densitometry analysis was performed using a GS-800 Calibrated Densitometer (Bio-Rad, Hercules, CA, USA). Western blot analysis was repeated three times, including four samples of each group in each membrane. 

### 4.8. Statistical Analysis

The sigma Plot software (version 14.5, Leighton Buzzard, UK) was used to analyze data by one-way or two-way ANOVA depending on the case, analyzing the genotype and/or the diet. Each group contained between 6 and 8 individuals (N number). *p* < 0.05 was assumed as statistically significant and is shown in the figures as *, ** or *** when *p* < 0.05, *p* < 0.01 and *p* < 0.001, respectively.

## Figures and Tables

**Table 1:** Fatty acid composition of each diet as percentage and mg/100 g. (n.d. = non-detectable; L = low ω-6/ω-3 ratio; SF = standard food; H = high ω-6/ω-3 ratio).

		L		SF		H	
**Total fat contain**		6.9 g/100g		4.6 g/100g		7.3 g/100g	
		relative %	mg/100g	relative %	mg/100g	relative %	mg/100g
**Saturated fatty acids**							
Total saturated fatty acids	SFA	12.7	871	15.1	654	8.3	579
Palmitic acid	C16:0	8	552	11.4	495	5.1	356
Stearic acid	C18:0	2.4	162	2.4	104	2.2	156
**Unsaturated fatty acids**							
Total unsaturated fatty acids	UFA	87.3	5858	84.9	3605	91.7	6287
Oleic acid	C18:1	40.5	2733	47	2002	55	3784
Monounsaturated fatty acids	MUFA	47.5	3201	47.8	2037	55.6	3825
Polyunsaturated fatty acids	PUFA	39.9	2657	37.1	1568	36.1	2463
**Omega 3 (ω3)**							
Omega 3	ω-3	9.4	615	2.8	119	1.4	94
Alpha-linolenic acid	ALA	2.9	193	2.8	116	1.2	80
Eicosapentaenoic acid	EPA	1.7	108	nd	nd	nd	nd
Docosohexaenoic acid	DHA	2.8	177	nd	nd	0.2	14
Docosapentaenoic acid	DPA	0.8	52	nd	nd	nd	nd
**Omega 6 (ω6)**							
Omega 6	ω-6	30.2	2025	34.3	1449	34.5	2356
Linoleic acid	LA	29.1	1952	34.2	1446	34.5	2356
**Ratio**							
LA/ALA		10		12.2		28.8	
ω -6/ω -3		3.2		12.3		24.6	
C18:1/C16:0		5.1		4.1		10.8	
SFA/ω-3		1.4		5.4		5.9	
C16:0/SFA		0.6		0.8		0.6	
C16:0/ALA		2.8		4.1		4.3	
PUFA/ALA		13.8		13.3		30.1	
SFA/ALA		4.4		5.4		6.9	

## Supplementary Materials

The following are available online at https://www.mdpi.com/article/10.3390/ijms222010907/s1.

## References

[1] Alzheimer A. (1907). Über eine eigenartige Erkrankung der Hirnrinde. Allg Z Psychiatr. Psych. Gerichtl. Med..

[2] Selkoe D.J. (2002). Alzheimer’s Disease Is a Synaptic Failure. Science.

[3] Gibson G.E., Huang H.M. (2005). Oxidative stress in Alzheimer’s disease. Neurobiol Aging.

[4] Hardy J., Selkoe D.J. (2002). The amyloid hypothesis of Alzheimer’s disease: Progress and problems on the road to therapeutics. Science.

[5] Mullan M., Crawford F., Axelman K., Houlden H., Lilius L., Winblad B., Lannfelt L. (1992). A pathogenic mutation for probable Alzheimer’s disease in the APP gene at the N-terminus of beta-amyloid. Nat Genet..

[6] Levy-Lahad E., Wasco W., Poorkaj P., Romano D.M., Oshima J., Pettingell W.H., E Yu C., Jondro P.D., Schmidt S.D., Wang K. (1995). Candidate gene for the chromosome 1 familial Alzheimer’s disease locus. Science.

[7] Sherrington R., Froelich S., Sorbi S., Campion D., Chi H., Rogaeva E.A., Levesque G., Rogaev E.I., Lin C., Liang Y. (1996). Alzheimer’s disease associated with mutations in presenilin 2 is rare and variably penetrant. Hum. Mol. Genet..

[8] Ettcheto M., Petrov D., Pedrós I., de Lemos L., Pallàs M., Alegret M., Laguna J.C., Folch J., Camins A. (2015). Hypercholesterolemia and neurodegeneration. Comparison of hippocampal phenotypes in LDLr knockout and APPswe/PS1dE9 mice. Exp. Gerontol..

[9] Dar T., Sheikh I., Ganie S., Ali R., Singh L., Gan S.H., Kamal M.A., Zargar M. (2014). Molecular Linkages Between Diabetes and Alzheimer’s Disease: Current Scenario and Future Prospects. CNS Neurol. Disord.-Drug Targets.

[10] Park S.H., Kim J.H., Choi K.H., Jang Y.J., Bae S.S., Choi B.T., Shin H.K. (2013). Hypercholesterolemia accelerates amyloid beta-induced cognitive deficits. Int. J. Mol. Med..

[11] Ricciarelli R., Canepa E., Marengo B., Marinari U.M., Poli G., Pronzato M.A., Domenicotti C. (2012). Cholesterol and Alzheimer’s disease: A still poorly understood correlation. IUBMB Life.

[12] Morris M.C., Evans D.A., Bienias J.L., Tangney C.C., Bennett D.A., Wilson R.S., Aggarwal N., Schneider J. (2003). Consumption of Fish and n-3 Fatty Acids and Risk of Incident Alzheimer Disease. Arch. Neurol..

[13] Scarmeas N., Stern Y., Tang M.-X., Mayeux R., Luchsinger J. (2006). Mediterranean diet and risk for Alzheimer’s disease. Ann. Neurol..

[14] Kao Y., Ho P.C., Tu Y.K., Jou I.M., Tsai K.J. (2020). Lipids and Alzheimer’s Disease. Int. J. Mol. Sci..

[15] Crivelli S.M., Giovagnoni C., Virsseren L., Scheithauer A.L., de Wit N., den Hoedt S., Losen M., Mulder M.T., Walter J., de Vries H.E. (2020). Sphingolipids in Alzheimer’s disease, how can we target them?. Adv. Drug Deliv. Rev..

[16] Bourre J.M. (1989). Nature, origin and role of fatty acids of the nervous system: An essential fatty acid, an alpha-linolenic acid, changing the structure and the cerebral function. Bull. l’Academie Natl. Med..

[17] Cervera M.A.R., Valenzuela R., Hernandez-Rodas M.C., Barrera C., Espinosa A., Marambio M., Valenzuela A. (2016). Vegetable oils rich in alpha linolenic acid increment hepatic n-3 LCPUFA, modulating the fatty acid metabolism and antioxidant response in rats. Prostaglandins Leukot. Essent. Fat. Acids.

[18] Sambra V., Echeverria F., Valenzuela A., Chouinard-Watkins R., Valenzuela R. (2021). Docosahexaenoic and Arachidonic Acids as Neuroprotective Nutrients throughout the Life Cycle. Nutrients.

[19] Joint F.A.O. (2010). Fats and fatty acids in human nutrition. Report of an expert consultation. FAO Food Nutr Pap..

[20] Cholewski M., Tomczykowa M., Tomczyk M. (2018). A Comprehensive Review of Chemistry, Sources and Bioavailability of Omega-3 Fatty Acids. Nutrients.

[21] Crawford M.A., Broadhurst C.L. (2012). The role of docosahexaenoic and the marine food web as determinants of evolution and hominid brain development: The challenge for human sustainability. Nutr. Health.

[22] Mazza M., Pomponi M., Janiri L., Bria P., Mazza S. (2007). Omega-3 fatty acids and antioxidants in neurological and psychiatric diseases: An overview. Prog. Neuro-Psychopharmacology Biol. Psychiatry.

[23] Catalan J., Moriguchi T., Slotnick B., Murthy M., Greiner R.S., Salem N. (2002). Cognitive deficits in docosahexaenoic acid-deficient rats. Behav. Neurosci..

[24] Ikemoto A., Ohishi M., Sato Y., Hata N., Misawa Y., Fujii Y., Okuyama H. (2001). Reversibility of n-3 fatty acid deficiency-induced alterations of learning behavior in the rat: Level of n-6 fatty acids as another critical factor. J. Lipid Res..

[25] Wiesmann M., Zerbi V., Jansen D., Haast R., Lütjohann D., Broersen L.M., Heerschap A., Kiliaan A.J. (2016). A Dietary Treatment Improves Cerebral Blood Flow and Brain Connectivity in Aging apoE4 Mice. Neural Plast..

[26] Lim G.P., Calon F., Morihara T., Yang F., Teter B., Ubeda O., Jr N.S., Frautschy S.A., Cole G.M. (2005). A Diet Enriched with the Omega-3 Fatty Acid Docosahexaenoic Acid Reduces Amyloid Burden in an Aged Alzheimer Mouse Model. J. Neurosci..

[27] Perez S.E., He B., Muhammad N., Oh K.J., Fahnestock M., Ikonomovic M.D., Mufson E.J. (2011). Cholinotrophic basal forebrain system alterations in 3xTg-AD transgenic mice. Neurobiol. Dis..

[28] Arsenault D., Julien C., Tremblay C., Calon F. (2011). DHA improves cognition and prevents dysfunction of entorhinal cortex neurons in 3xTg-AD mice. PLoS ONE.

[29] Herrera J.L., Ordoñez-Gutierrez L., Fabrias G., Casas J., Morales A., Hernández G., Acosta N.G., Rodriguez C., Prieto-Valiente L., Garcia-Segura L.M. (2019). Ovarian Hormone-Dependent Effects of Dietary Lipids on APP/PS1 Mouse Brain. Front. Aging Neurosci..

[30] Schwarz J.M., Sholar P.W., Bilbo S. (2011). Sex differences in microglial colonization of the developing rat brain. J. Neurochem..

[31] Yanguas-Casás N., Crespo-Castrillo A., de Ceballos M.L., Chowen J., Azcoitia I., Arevalo M.-A., Garcia-Segura L.M. (2017). Sex differences in the phagocytic and migratory activity of microglia and their impairment by palmitic acid. Glia.

[32] Morselli E., Criollo A., Rodriguez-Navas C., Clegg D.J. (2015). Chronic High Fat Diet Consumption Impairs Metabolic Health of Male Mice. Inflamm. Cell Signal..

[33] Argente-Arizón P., Díaz F., Ros P., Barrios V., Tena-Sempere M., García-Segura L.M., Argente J., A Chowen J. (2017). The Hypothalamic Inflammatory/Gliosis Response to Neonatal Overnutrition Is Sex and Age Dependent. Endocrinology.

[34] Herrera J.L., Ordoñez-Gutierrez L., Fabrias G., Casas J., Morales A., Hernández G., Acosta N.G., Rodriguez C., Prieto-Valiente L., Garcia-Segura L.M. (2018). Ovarian Function Modulates the Effects of Long-Chain Polyunsaturated Fatty Acids on the Mouse Cerebral Cortex. Front. Cell. Neurosci..

[35] Koivisto H., Grimm M., Rothhaar T.L., Berkecz R., Lütjohann D., Giniatullina R., Takalo M., Miettinen P.O., Lahtinen H.-M., Giniatullin R. (2013). Special lipid-based diets alleviate cognitive deficits in the APPswe/PS1dE9 transgenic mouse model of Alzheimer’s disease independent of brain amyloid deposition. J. Nutr. Biochem..

[36] Dyall S.C. (2017). Interplay Between n-3 and n-6 Long-Chain Polyunsaturated Fatty Acids and the Endocannabinoid System in Brain Protection and Repair. Lipids.

[37] Farooqui A. (2009). Beneficial Effects of Fish. Oil on Human Brain.

[38] Green K.N., Martinez-Coria H., Khashwji H., Hall E.B., Yurko-Mauro K.A., Ellis L., LaFerla F.M. (2007). Dietary docosahexaenoic acid and docosapentaenoic acid ameliorate amyloid-beta and tau pathology via a mechanism involving presenilin 1 levels. J. Neurosci..

[39] Perez S.E., Berg B.M., Moore K.A., He B., Counts S.E., Fritz J.J., Hu Y., Lazarov O., Lah J.J., Mufson E.J. (2010). DHA diet reduces AD pathology in young APPswe/PS1 Delta E9 transgenic mice: Possible gender effects. J. Neurosci. Res..

[40] Samieri C., Lorrain S., Buaud B., Vaysse C., Berr C., Peuchant E., Cunnane S.C., Barberger-Gateau P. (2013). Relationship between diet and plasma long-chain n-3 PUFAs in older people: Impact of apolipoprotein E genotype. J. Lipid Res..

[41] Olivera-Perez H.M., Lam L., Dang J., Jiang W., Rodriguez F., Rigali E., Weitzman S., Porter V., Rubbi L., Morselli M. (2017). Omega-3 fatty acids increase the unfolded protein response and improve amyloid-beta phagocytosis by macrophages of patients with mild cognitive impairment. FASEB J..

[42] Hjorth E., Zhu M., Cortés-Toro V., Vedin I., Palmblad J., Cederhom T., Freund-Levi Y., Wahlund L., Basun H., Eriksdotter M. (2013). Omega-3 fatty acids enhance phagocytosis of Alzheimer’s disease-related amyloid-beta42 by human microglia and decrease inflammatory markers. J. Alzheimers Dis..

[43] Wang J., Tanila H., Puoliväli J., Kadish I., van Groen T. (2003). Gender differences in the amount and deposition of amyloidbeta in APPswe and PS1 double transgenic mice. Neurobiol. Dis..

[44] Barron A.M., Rosario E.R., Elteriefi R., Pike C.J. (2013). Sex-specific effects of high fat diet on indices of metabolic syndrome in 3xTg-AD mice: Implications for Alzheimer’s disease. PLoS ONE.

[45] Arcones A., Cruces-Sande M., Ramos P., Mayor F., Murga C. (2019). Sex Differences in High Fat Diet-Induced Metabolic Alterations Correlate with Changes in the Modulation of GRK2 Levels. Cells.

[46] Wells A., Barrington W.T., Dearth S., May A., Threadgill D.W., Campagna S.R., Voy B.H. (2018). Tissue Level Diet. and Sex.-by-Diet. Interactions Reveal Unique Metabolite and Clustering Profiles Using Untargeted Liquid Chromatography-Mass Spectrometry on Adipose, Skeletal Muscle, and Liver Tissue in C57BL6/J. Mice. J. Proteome Res..

[47] Hooijmans C.R., Rutters F., Dederen P., Gambarota G., Veltien A., van Groen T., Broersen L., Lütjohann D., Heerschap A., Tanila H. (2007). Changes in cerebral blood volume and amyloid pathology in aged Alzheimer APP/PS1 mice on a docosahexaenoic acid (DHA) diet or cholesterol enriched Typical Western Diet (TWD). Neurobiol. Dis..

[48] Barberger-Gateau P., Letenneur L., Deschamps V., Peres K., Dartigues J.-F., Renaud S. (2002). Fish, meat, and risk of dementia: Cohort study. BMJ.

[49] Calon F., Lim G.P., Yang F., Morihara T., Teter B., Ubeda O., Rostaing P., Triller A., Salem N., Ashe K.H. (2004). Docosahexaenoic Acid Protects from Dendritic Pathology in an Alzheimer’s Disease Mouse Model. Neuron.

[50] Cederholm T., Palmblad J. (2010). Are omega-3 fatty acids options for prevention and treatment of cognitive decline and dementia?. Curr. Opin. Clin. Nutr. Metab. Care.

[51] Yassine H.N., Feng Q., Azizkhanian I., Rawat V., Castor K., Fonteh A.N., Harrington M., Zheng L., Reed B.R., DeCarli C. (2016). Association of Serum Docosahexaenoic Acid With Cerebral Amyloidosis. JAMA Neurol..

[52] Panchal M., Gaudin M., Lazar A.N., Salvati E., Rivals I., Ayciriex S., Dauphinot L., Dargère D., Auzeil N., Masserini M. (2014). Ceramides and sphingomyelinases in senile plaques. Neurobiol. Dis..

[53] Puglielli L., Ellis B.C., Saunders A.J., Kovacs D.M. (2003). Ceramide stabilizes beta-site amyloid precursor protein-cleaving enzyme 1 and promotes amyloid beta-peptide biogenesis. J. Biol. Chem..

[54] Patil S., Melrose J., Chan C. (2007). Involvement of astroglial ceramide in palmitic acid-induced Alzheimer-like changes in primary neurons. Eur. J. Neurosci..

[55] He X., Huang Y., Li B., Gong C.-X., Schuchman E.H. (2010). Deregulation of sphingolipid metabolism in Alzheimer’s disease. Neurobiol. Aging.

[56] Filippov V., Song M.A., Zhang K., Vinters H.V., Tung S., Kirsch W.M., Yang J., Duerksen-Hughes P.J. (2012). Increased Ceramide in Brains with Alzheimer’s and Other Neurodegenerative Diseases. J. Alzheimer’s Dis..

[57] Ellis B., Hye A., Snowden S.G. (2015). Metabolic Modifications in Human Biofluids Suggest the Involvement of Sphingolipid, Antioxidant, and Glutamate Metabolism in Alzheimer’s Disease Pathogenesis. J. Alzheimers Dis..

[58] Bandaru V.V.R., Troncoso J., Wheeler D., Pletnikova O., Wang J., Conant K., Haughey N.J. (2009). ApoE4 disrupts sterol and sphingolipid metabolism in Alzheimer’s but not normal brain. Neurobiol. Aging.

[59] Cutler R.G., Kelly J., Storie K., Pedersen W.A., Tammara A., Hatanpaa K., Troncoso J.C., Mattson M.P. (2004). Involvement of oxidative stress-induced abnormalities in ceramide and cholesterol metabolism in brain aging and Alzheimer’s disease. Proc. Natl. Acad. Sci. USA.

[60] Sugimoto M., Shimizu Y., Yoshioka T., Wakabayashi M., Tanaka Y., Higashino K., Numata Y., Sakai S., Kihara A., Igarashi Y. (2015). Histological analyses by matrix-assisted laser desorption/ionization-imaging mass spectrometry reveal differential localization of sphingomyelin molecular species regulated by particular ceramide synthase in mouse brains. Biochim. Biophys. Acta (BBA)-Mol. Cell Biol. Lipids.

[61] Zhao L., Spassieva S.D., Jucius T.J., Shultz L.D., Shick H.E., Macklin W.B., Hannun Y.A., Obeid L., Ackerman S.L. (2011). A Deficiency of Ceramide Biosynthesis Causes Cerebellar Purkinje Cell Neurodegeneration and Lipofuscin Accumulation. PLoS Genet..

[62] Laviad E.L., Albee L., Pankova-Kholmyansky I., Epstein S., Park H., Merrill A.H., Futerman A.H. (2008). Characterization of ceramide synthase 2: Tissue distribution, substrate specificity, and inhibition by sphingosine 1-phosphate. J. Biol. Chem..

[63] Babenko N.A., Semenova Y.A. (2010). Effects of long-term fish oil-enriched diet on the sphingolipid metabolism in brain of old rats. Exp. Gerontol..

[64] Song Y., Cong P., Lu L., Wang Y., Tang Q., Zhang H., Xu J., Xue C. (2017). Effects of dietary glucocerebrosides from sea cucumber on the brain sphingolipid profiles of mouse models of Alzheimer’s disease. Food Funct..

[65] Oshida K., Shimizu T., Takase M., Tamura Y., Shimizu T., Yamashiro Y. (2003). Effects of Dietary Sphingomyelin on Central Nervous System Myelination in Developing Rats. Pediatr. Res..

[66] Allende M.L., Proia R.L. (2014). Simplifying complexity: Genetically resculpting glycosphingolipid synthesis pathways in mice to reveal function. Glycoconj. J..

[67] Barrier L., Ingrand S., Damjanac M., Bilan A.R., Hugon J., Page G. (2007). Genotype-related changes of ganglioside composition in brain regions of transgenic mouse models of Alzheimer’s disease. Neurobiol. Aging.

[68] Fabelo N., Martín M.V., Marín R., Santpere G., Aso E., Ferrer I., Díaz M. (2012). Evidence for Premature Lipid Raft Aging in APP/PS1 Double-Transgenic Mice, a Model of Familial Alzheimer Disease. J. Neuropathol. Exp. Neurol..

[69] Whiley L., Sen A., Heaton J., Proitsi P., García-Gómez D., Leung R., Smith N., Thambisetty M., Kloszewska I., Mecocci P. (2013). Evidence of altered phosphatidylcholine metabolism in Alzheimer’s disease. Neurobiol. Aging.

[70] Kennedy M.A., Moffat T.C., Gable K., Ganesan S., Niewola-Staszkowska K., Johnston A., Nislow C., Giaever G., Harris L.J., Loewith R. (2016). A Signaling Lipid Associated with Alzheimer’s Disease Promotes Mitochondrial Dysfunction. Sci. Rep..

[71] Brenna J.T., Salem N., Sinclair A.J., Cunnane S.C. (2009). Alpha-Linolenic acid supplementation and conversion to n-3 long-chain polyunsaturated fatty acids in humans. Prostaglandins Leukot. Essent. Fat. Acids.

[72] Xu N., Li A.-D., Ji L.-L., Ye Y., Wang Z.-Y., Tong L. (2019). miR-132 regulates the expression of synaptic proteins in APP/PS1 transgenic mice through C1q. Eur. J. Histochem..

[73] Xu Y.-J., Mei Y., Qu Z.-L., Zhang S.-J., Zhao W., Fang J.-S., Wu J., Yang C., Liu S.-J., Fang Y.-Q. (2018). Ligustilide Ameliorates Memory Deficiency in APP/PS1 Transgenic Mice via Restoring Mitochondrial Dysfunction. BioMed Res. Int..

[74] Zhang Y., Huang L.-J., Shi S., Xu S.-F., Wang X.-L., Peng Y. (2016). L-3-n-butylphthalide Rescues Hippocampal Synaptic Failure and Attenuates Neuropathology in Aged APP/PS1 Mouse Model of Alzheimer’s Disease. CNS Neurosci. Ther..

[75] Pedrós I., Petrov D., Allgaier M., Sureda F.X., Barroso E., Beas-Zarate C., Auladell C., Pallàs M., Vázquez-Carrera M., Casadesus G. (2014). Early alterations in energy metabolism in the hippocampus of APPswe/PS1dE9 mouse model of Alzheimer’s disease. Biochim. Biophys. Acta (BBA)-Mol. Basis Dis..

[76] Liu B., Kou J., Li F., Huo D., Xu J., Zhou X., Meng D., Ghulam M., Artyom B., Gao X. (2020). Lemon essential oil ameliorates age-associated cognitive dysfunction via modulating hippocampal synaptic density and inhibiting acetylcholinesterase. Aging.

[77] Tao G., Luo Y., Xue Q., Li G., Tan Y., Xiao J., Yu B. (2016). Docosahexaenoic Acid Rescues Synaptogenesis Impairment and Long-Term Memory Deficits Caused by Postnatal Multiple Sevoflurane Exposures. BioMed Res. Int..

[78] Sophocleous A., Idris A. (2019). Ovariectomy/Orchiectomy in Rodents. Bone Research Protocols.

[79] Jankowsky J.L., Slunt H.H., Ratovitski T., Jenkins N.A., Copeland N.G., Borchelt D.R. (2001). Co-expression of multiple transgenes in mouse CNS: A comparison of strategies. Biomol. Eng..

[80] Ordoñez-Gutierrez L., Antón M., Wandosell F. (2015). Peripheral amyloid levels present gender differences associated with aging in AbPP/PS1 mice. J. Alz. Dis..

[81] Cingolani F., Casasampere M., Sanllehí P., Casas J., Fàbrias G. (2014). Inhibition of dihydroceramide desaturase activity by the sphingosine kinase inhibitor SKII. J. Lipid. Res..

[82] Garanto A., Mandal N.A., Egido-Gabás M., Marfany G., Fabrias G., Anderson R.E., Casas J., Gonzàlez-Duarte R. (2013). Specific sphingolipid content decrease in Cerkl knockdown mouse retinas. Exp. Eye Res..

