# Supplementary material for: Diets with Higher ω-6/ω-3 Ratios Show Differences in Ceramides and Fatty Acid Levels Accompanied by Increased Amyloid-Beta in the Brains of Male APP/PS1 Transgenic Mice
Source: Int J Mol Sci. 2021 Oct 9;22(20):10907. doi: 10.3390/ijms222010907 (PMC8535881; doi:10.3390/ijms222010907)
Supplement: Supplementary file 1 [file ijms-22-10907-s001.zip › ijms-1336612-supplementary.pdf]

Supplementary Table S1. Total fat content of the three diets: L, H and SF. (n.d.= non-detectable, L= low ω-6/ω-3 ratio, SF = standard food, H= high ω-6/ω-3 ratio).

		L		SF		H	
**Total fat contain**		6.9g/100g		4.6g/100g		7.3g/100g	
		**relative %**	**mg/100g**	**relative %**	**mg/100g**	**relative %**	**mg/100g**
Myristic acid	C14:0	1.3	95	0.1	<10	0.1	<10
Myristoleic acid	C14:1	nd	nd	nd	nd	nd	nd
Pentadecylic acid	C15:0	0.2	<10	nd	nd	nd	nd
Palmitic acid	C16:0	8	552	11.4	495	5.1	356
Palmitoleic acid	C16:1	1.6	109	0.2	<10	0.1	<10
Margaric acid	C17:0	0.1	<10	nd	nd	0.1	<10
Stearic acid	C18:0	2.4	162	2.4	104	2.2	156
Oleic acid	C18:1	38.2	2582	46	1961	53.6	3685
Linoleic acid	C18:2	29.1	1952	34.2	1446	34.5	2356
Alpha-Linolenic acid	C18:3	2.9	193	2.8	116	1.2	80
Stearidonic acid	C18:4	0.4	26	nd	nd	nd	nd
Nonadecylic acid	C19:0	nd	nd	nd	nd	nd	nd
Arachidic acid	C20:0	0.4	24	0.3	12	0.4	26
Gadoleic acid	C20:1	2.7	180	0.6	25	0.3	23
Eicosadienoic acid	C20:2	0.7	47	0.1	<10	nd	nd
Eicosatrienoic acid	C20:3	0.5	32	0.1	<10	nd	nd
Arachidonic acid	C20:4	0.2	12	nd	nd	nd	nd
Eicosapentaenoic acid	C20:5	1.7	108	nd	nd	nd	nd
Heneicosylic acid	C21:0	nd	nd	nd	nd	nd	nd
Behenic acid	C22:0	0.2	15	0.5	23	0.3	18
Erucic acid	C22:1	0.4	26	nd	nd	nd	nd
Docosadienoic acid	C22:2	nd	nd	nd	nd	nd	nd
Docosatrienoic acid	C22:3	nd	nd	nd	nd	nd	nd
Docosatetraenoic acid	C22:4	nd	nd	nd	nd	nd	nd
Docosapentaenoic acid	C22:5	0.8	52	nd	nd	nd	nd
Docosahexaenoic acid	C22:6	2.8	177	nd	nd	0.2	14
Tricosylic acid	C23:0	nd	nd	0.1	<10	nd	nd
Lignoceric acid	C24:0	0.1	<10	0.3	11	0.1	<10
Nervonic acid	C24:1	0.3	23	nd	nd	0.1	<10

Antibody	Host	Reference
Phospho-Synapsin Serine 9	Rabbit	#2311 Cell Signaling
P120	Mouse	#610133 BD Transduction Lab
PSD95	Rabbit	#3450 Cell Sabnaling
Synaptophysin (SY38)	Rabbit	#10701 Progen
Beta amyloid, 1-16(6E10) monoclonal antibody6e10	Mouse	#9331 Cell Signaling
GFAP	Rabbit	#Z0334Dako
BACE	Rabbit	#5606 Cell Signaling
PHF1	Mouse	Dr. Davies gift
GAPDH	Mouse	#47724 Santa Cruz Laboratories
Actin	Mouse	#A5441 Sigma-Aldrich
Vinculin	Mouse	#V9131 Sigma-Aldrich

Supplementary Table S2. Primary and secondary antibodies used.

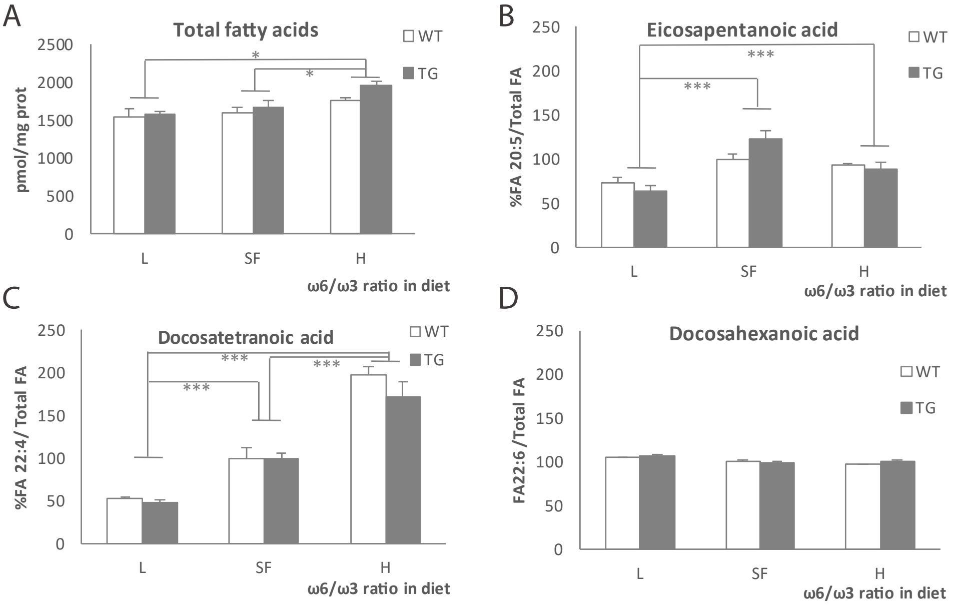

Supplementary Fig. S1. (**A**) Fatty acid (FA), (**B**) eicosapentaenoic acid (C20:5), (**C**) docosatetraenoic acid (C22:4) and (**D**) docosahexaenoic acid (C22:6) levels in the brains of transgenic (TG) and wild-type (WT) male mice after being fed with L, SF and H diets for 3 months. Results are shown as the medium ± SEM of the percentage with respect to total FA and represented with respect to ω-6/ω-3 ratio in the diet. *: $p<0.05$; ***: $p<0.001$. L=lower ω-6/ω-3 ratio, H= higher ω-6/ω-3 ratio, SF= standard food.

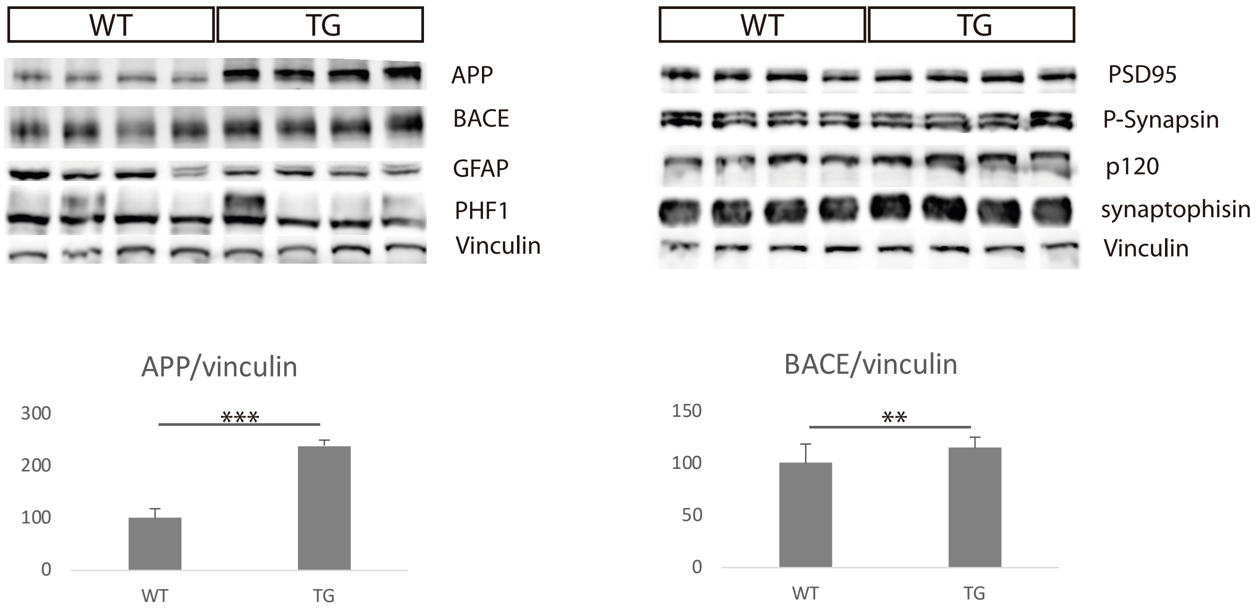

Supplementary Fig. S2. Synaptic and pathological markers in transgenic (TG) and wild-type (WT) mice. PSD95, phospho-synapsin, p120 and synaptophysin were checked as synaptic markers. APP, BACE, GFAP and PHF1 were checked as markers involved in the pathological process, Aβ accumulation and Tau phosphorylation. Results are shown as the medium ± SEM as the percentage respect to WT. **: $p<0.01$, ***: $p<0.001$.

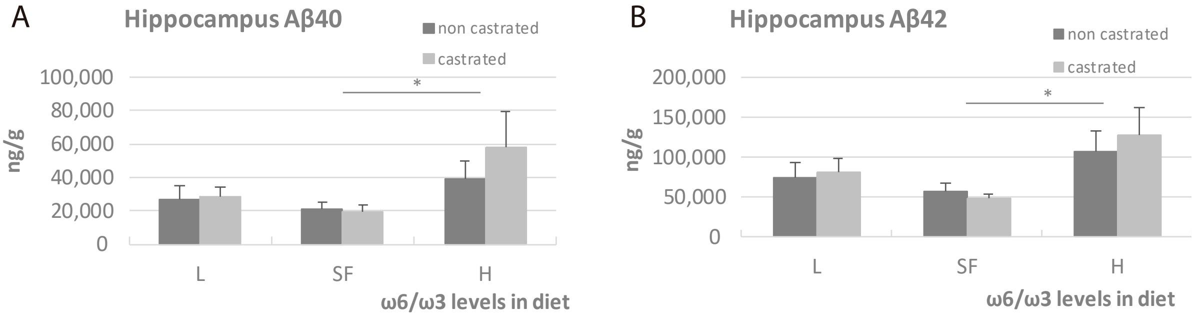

Supplementary Fig. S3. Aβ1-40 (**A**) and Aβ1-42 (**B**) levels in the hippocampus of transgenic male mice after being fed with L, SF and H diets for 3 months. The castrated group was operated on at two months-of-age, one month before the modified-diet was supplied. Data are represented with respect to ω-6/ω-3 ratio in the diet. Results are shown as the medium ± SEM. *: $p<0.05$.
